# Underlying Familial Factors for Aggressive Behavior in Romantic Relationships: A Systematic Review

**DOI:** 10.3390/ijerph19084485

**Published:** 2022-04-08

**Authors:** Shalini Munusamy, Sobana Jeyagobi, Isa Naina Mohamed, Jaya Kumar Murthy, Sheau Tsuey Chong, Hilwa Abdullah, Mohamamad Rahim Kamaluddin

**Affiliations:** 1 Centre for Research in Psychology and Human Well-Being, Faculty of Social Sciences and Humanities, Universiti Kebangsaan Malaysia, Bangi 43600, Malaysia; shalinim@utar.edu.my (S.M.); sobana.j17@gmail.com (S.J.); stchong@ukm.edu.my (S.T.C.); hilwa@ukm.edu.my (H.A.); 2 Department of Early Childhood Education, Faculty of Creative Industries, Universiti Tunku Abdul Rahman Sungai Long, Kajang 43000, Malaysia; 3 Department of Pharmacology, Faculty of Medicine, Universiti Kebangsaan Malaysia (The National University of Malaysia), Kuala Lumpur 56000, Malaysia; isanaina@ppukm.ukm.edu.my (I.N.M.); jayakumar@ukm.edu.my (J.K.M.)

**Keywords:** familial factors, family supports, family relationship, parenting, aggressive behavior, romantic relationship

## Abstract

Aggressive behavior in romantic relationship has serious effects, including both intra- and inter-personal issues. Aggressive behaviors in romantic relationships have been linked to underlying familial problems. While there have been previous reviews that studied on many interpersonal and dyadic implications of aggressive behavior in romantic relationships, there is nonetheless a lack of studies on the various components of familial factors for aggressive behavior in romantic relationships. The databases *Scopus*, *MEDLINE*, *Google Scholar*, and *SAGE Journals* were used to search for terms that are related to familial factors (family factor, family support, family relationship) as well as terms related to aggressive behavior in romantic relationships (aggression in romantic relationship, violence in intimate relationship). The articles considered for this review were original studies, samples, or subsamples of males or females who reported any underlying familial factors in childhood or adulthood that contributed to aggressive behavior in romantic relationship, and the studies must be written in English. This review has 27 papers that met the inclusion criteria. The findings from this review revealed the presence of inconsistent conclusions between familial factors and aggressive behavior in romantic relationships, with some studies failing to establish such links. These findings are reviewed with regards to the existing gaps in the literature as well as potential research options.

## 1. Introduction

Romantic relationship has a different meaning for each individual and is said to have an impact on people’s lives [1]. Romantic relationship issues have attracted the interest of many deep researchers into the field of social sciences, and the topic has even attracted the attention of politicians and local communities. Romantic relationships are often seen as one of the social problems that need to be addressed [2]. Aggressive behavior in romantic relationships is a serious public health issue that affects a large number of teenagers and adults. According to recent meta-analytic findings on the incidence of dating abuse by Wincentak, Connolly, and Card (2017) [3], 20% of 13- to 18-year-olds had been physically abused, with 14% of girls and 8% of boys reporting sexual violence. Aggression behavior in a romantic relationship is commonly referred to as dating violence (DV) or intimate partner violence (IPV) [3]. The Center for Disease Control and Prevention (2016) [4] defined psychological abuse as threats or injury to a partner’s sense of self-worth, such as name-calling, bullying, insulting, or seeking to isolate him or her from friends and family. Physical hostility might include pinching, striking, shoving, slapping, punching, or kicking. When a partner is pushed, coerced, or bullied into engaging in inappropriate sexual practices, this is known as sexual violence. Emotional abuse is also one of the most common forms of abuse in romantic relationships [5].

Aggressive behavior in romantic relationship is defined as aggression within a current or former intimate relationship with any physical violence, such as slapping, punching, and kicking; psychological aggression, such as yelling, embarrassing, and name-calling; sexual violence, such as rape, sexual assault, unwanted sexual attention, and sexual coercion; stalking, such as unwanted attention that causes fear or concern for one’s safety; or any combination of one or more aggression in a romantic relationship. 

Physical, sexual, psychological, or emotional aggression as well as stalking are all examples of dating violence [4]. In addition, Rahim et al. (2016) [6] stated that aggressive behavior is a criminogenic trait, which is often associated with various violent crimes including dating violence. The online dating scam is also another example of dating violence, which is also alarming because it has effects on individuals such as financial loss and loss of a relationship, which involve severe emotional and psychological suffering [7]. Besides that, another type of aggressive behavior in romantic relationships is called relational aggression. Kokkinos (2015) [8] defined the term “romantic relational aggression” as the practice of injuring, using rumors to manipulate romantic partners’ social ties, love withdrawal, and jealousy induction. Depressive disorders, alcohol-related difficulties, and physical health difficulties were all linked to perpetrating or being a victim of romantic relationship aggression [9]. 

Romantic relationship violence is a particularly dangerous form of aggression because of its high prevalence and covert nature. Although most studies on relational violence focus on peer interactions, it can also be aimed towards a romantic partner [10]. Aggressive behavior in romantic relationship is not a new phenomenon. Many studies have been performed by researchers. Furthermore, many models and theories have been formed to understand the concept of aggressive behavior in romantic relationships. For example, O’Keefe’s (1995) [11] model looked at behavioral aspects where aggressive behavior more comprehensively encompassed the individual and the environment, namely personality, sociological, and psychological. Theory of Tension (Strain Theory) by Agnew (1992) [12] also says an individual will experience negative emotions, such as frustration and being aggressive, when he or she is not able to achieve goals through proper and conventional means. This causes individuals to choose an alternative path by violating the norms of life.

A study conducted by Tussey, Tyler, and Simons (2018) [13] on parents’ attachment style and dating violence shows that parenting style is linked to perpetration in intimate relationships and that this normalizing of violence is linked to future dating situations, including violence. Familial factors can be defined as a term that is used to indicate a component or condition that is seen in a family and accounts for a variety of diseases, ailments, or features that can have a variety of consequences on individuals. Many researchers have attempted to explore the risk factors involved with aggression behavior in romantic relationships in order to anticipate individuals who are most at risk of aggression behavior in romantic relationships and, as a result, to design ways to prevent aggression behavior in romantic relationships or minimize its consequences.

The environment is a critical aspect in one’s development, and families are the most frequently considered environmental influences [14,15]. In addition, to explore how familial factors are linked to young adults’ aggressive behavior in romantic relationships, researchers have used a variety of theoretical perspectives, including social learning theory, the background situational model of dating violence, and the antisocial orientation perspective. According to social learning theory, aggression directed towards others is taught through observational learning from one’s social context [16]. Furthermore, early exposure to specific types of family violence and abuse is linked to the development of specific forms of aggression later in life [17,18].

Several reviews of various kinds have been conducted over the years to investigate the link between the two conceptions. In a review of risk variables for dating victimization in young people, Vezina and Hebert (2007) [19] discovered that watching or personally experiencing familial violence is associated to victimization in dating relationships. Bonding and closeness with parents create a sense of belonging and self-worth, making a young person less likely to engage in criminal behavior or tolerate abusive spouses [20]. Successful discipline, setting of boundaries, open communication, conflict resolution, and understanding of the youth’s activities have all been linked to reduced risks of dating abuse [21]. Participation of parents and their comprehension towards young adults in an abusive relationship may help them feel less isolated through assistance and support. Furthermore, few reviews on dating violence and risk factors were conducted [22,23,24]. 

According to this review, individual risk factors, family risk factors, and other factors, such as cognitive factors and interactional partners, were significantly associated with dating violence. While previous studies have linked family factors to dating violence among young adults [14,25,26], little effort has been made to simultaneously recognize the significantly influence empirically linked to aggressive behavior in romantic relationships. Thus far, very few reviews have attempted to investigate aggression behavior in romantic relationships while simultaneously situating their findings on family factors. Similarly, past research has failed to investigate the interplay between these factors that may reinforce or protect this demographic from dating violence.

We found a gap in the research after exploring existing reviews discussing the relationship between familial factors and aggression behavior in romantic relationships. There are very few reviews that have examined studies that explored the relationship between familial factors and aggression behavior in romantic relationships in all its manifestations, adopted a systematic approach, or it considered studies conducted among male and female samples. As a result, the goal of this systematic review is to compile and synthesize findings from several studies that have looked into the relationship between underlying familial factors and aggression behavior in romantic relationships, taking into account both male and female samples and not only couples. 

It is critical for therapists and researchers to have a better understanding of the relationships between the two domains in order to develop particular programs as well as to prevent and intervene appropriately for both perpetrators and victims. Overall, the data imply that adolescents with positive familial factors are more competent and less aggressive in adult romantic life. However, it is unclear how familial factors are linked to specific aggressive behaviors in romantic relationship functioning. This research will help to disperse some light on the conflicting findings across research, covering aspects regarding underlying familial factors and aggressive behavior in romantic relationship functioning.

## 2. Materials and Methods

**Figure 1:**
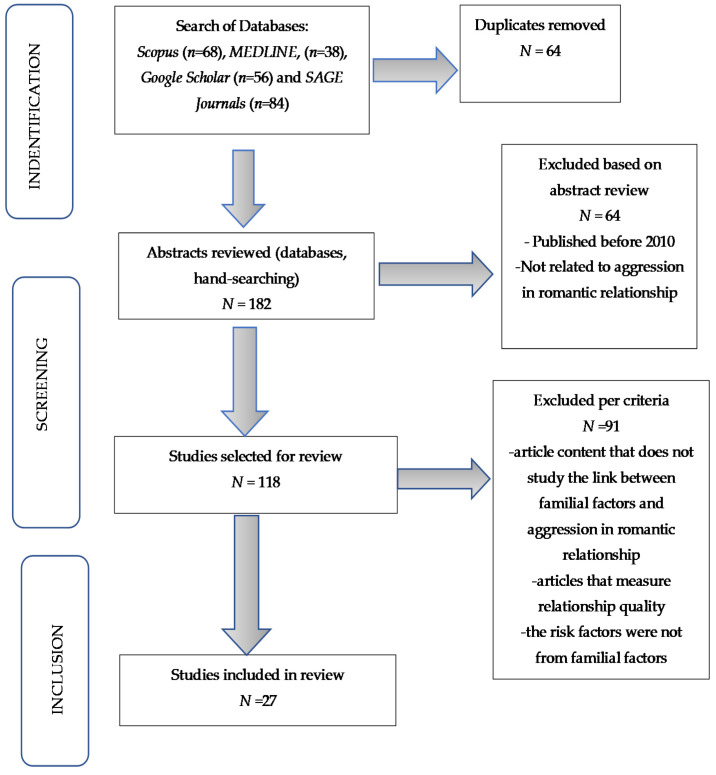
Flowchart of article identification, screening, and inclusion.

This systematic review uses the PRISMA (Preferred Reporting Items for Systematic reviews and Meta-Analyzes) criteria, and the PRSIMA flowchart was utilized to summarize the search procedure [27]. PRISMA is an upgraded version of the QUAROM guideline, which comprises a checklist and flowchart with 27 components. According to Sierra-Correa and Cantera Kintz (2015) [27], PRISMA has three distinct advantages: defining precise research questions in order to undertake systematic highlights, selecting inclusive and exclusive criteria, and reviewing a large database in a timely manner. PRISMA was used to discover previous studies on the association between underlying familial factors for aggressive behavior in romantic relationships in this systematic review. *Scopus*, *Google Scholar*, *MEDLINE*, and *SAGE Journals* were the four databases used in our search. Three steps are involved in systematic review process. The first step is identification. Next is screening, and the last part is the inclusion process (refer to Figure 1).

The first phase involves identifying the keywords to be used for search strategy process. Depending on past studies, similar keywords and keywords related to underlying familial factors and aggressive behavior in romantic relationships were used. The keyword list was reviewed and corrected, for example, the addition of different spelling variations. Table 1 presents the final version of the keyword search list. The primary focus of this systematic review is to identify empirical quantitative studies that explored the connection between underlying familial factors with aggressive behavior in romantic relationships. Several criteria were taken into account as a means for finding criteria, and we have presented these criteria in Table 2. In this research, we searched the publication dates limited to 2010 and 2021 (articles that were published in the past eleven years) so that our review could be based on the most recent literature on information retrieval and synthesis in the digital age.

The *Scopus*, *MEDLINE*, *Google Scholar*, and *SAGE Journals* databases were searched for studies from 4 May 2021 to 30 May 2021. Figure 1 shows the total number of articles that were discovered, the number of remaining articles after removing duplicates, the number of articles that did not meet the inclusion requirements, as well as the articles that were chosen for further study. Initially, there were 246 papers. However, upon screening the reference list, which was performed by one independent reviewer (the first author), 182 articles were discovered using the search criteria across all four databases. The title and abstract of each paper were evaluated for relevance during the first screening step, and 118 titles were removed. The complete text of the studies that were judged acceptable was next assessed for eligibility according to their methodology and findings. Several manuscripts were eliminated for various reasons after reading the entire article. Manuscripts that did not specify the term familial variables, manuscripts that were review manuscripts as secondary sources, and manuscripts that merely measured the relationship quality were among those eliminated. There were 27 manuscripts that were included and found eligible for examination. The PRISMA flow diagram is shown in Figure 1.

## 3. Results

See Figure 1 for the flow diagram of study selection.

### 3.1. Measurement of Aggressive Behavior in Romantic Relationship

Common measures used in samples that involved adolescents include the Conflict Tactic Scale, Conflict in Relationships Scale, and the Conflict in Adolescent Dating Relationships Inventory. A plethora of studies combined physical and dating violence as common measures. However, aggression among adolescents as rarely addressed in the majority of research. Studies on aggressive behavior in romantic relationships relied primarily on the Conflict Tactics Scale (CTS) [18] in some form, or selected questions from the measure were used to measure aggressive behavior in romantic relationship [14,28,29,30,31,32,33,34,35,36,37]. Among the studies, the most frequent measures used after the CTS were the Conflict in Relationship Scale [28,37,38,39,40,41], the Aggression Questionnaire (AQ) [42,43,44], Aggression and Social Behavior Measure (SRASMB) [44,45], and Partner-Directed Cyber Aggression [46]. Relational aggression and victimization in romantic relationships were rarely addressed in a significant amount of research [47,48].

### 3.2. Synthesized Findings

A total of twenty-seven studies were selected for review. All the manuscripts that were reviewed studied different types of relationships. Five manuscripts studied the relationship between attachment with parents and aggressive behavior in romantic relationships; five out of twenty-nine manuscripts measured attachment style with parents as part of their study, accounting for 17.24% of the total manuscripts [34,36,39,41,44]. Six out of twenty-seven (20.68%) explored the relationship between family violence and aggressive behavior in romantic relationships. Three studies (10.3%) reviewed the association between aggressive behavior and parental divorce in romantic relationships [36,49,50]. Two studies (6.9%) of the found manuscripts reviewed the association between aggressive behavior and parental style in romantic relationship [45,51]. One study defined family social structure as the social position or status of the home within the prevailing societal stratification [33]. 

*The Exposure of Intimate Partner Violence in Family of Origin*. Social learning theory states that exposure towards violence in the origin family or witnessing violence conducted by parents as a child is an extensively studied risk factor for aggressive behavior in romantic relationships. A significantly long research time is necessary in order to conduct such a method, which also is fortunately true for other related familial risk factors. Sadly, this issue is rarely solved by utilizing a fully prospective design. Vezina, Hebert, Poulin, Lavoie, Vitaro, and Tremblay (2015) [36] discovered that past related parental violence was linked with a higher likeliness of girls being victimized psychologically, sexually, or physically in their romantic life regardless of the phase, including adolescence, early adulthood, or both developmental phases. Similarly, Laporte et al. (2011) [30] discovered that, depending on gender and risk levels, teenagers carry negative childhood memories of family violence into their romantic relationships in diverse ways. Female adolescents who had been abused by either of their parents are more prone to experience re-victimization rather than aggression in their love relationships. High-risk adolescent boys who were victimized as a child are more likely to be aggressive toward their girlfriends, especially if their father had chastised them forcefully. Overall, there is a low to moderately significant association between seeing parental violence or family aggression and subsequent aggressive behavior perpetration or victimization in romantic relationships. Nonetheless, the evidence obtained is based on retrospective reporting, and some data imply that other proximal characteristics, such as the individual’s aggressive behavior and adult adjustment, may mitigate the relationship [14,26,29,38,40].

*Parenting.* Parental surveillance was shown to prevent children from a range of problem behaviors during adolescence, and has been studied in relation to aggressive behavior in romantic relationships. One family component that has long-term effects on young adult love connections is effective parenting qualities, such as inductive reasoning and consistent and moderate limit setting [52,53]. According to Xia et al. (2018) [37], young adults that grew up with a positive family ambience and efficient parenting approaches had better decision-making skills and were less prone to participate in interpersonal violence among young adult relationships. Furthermore, contrary to what was expected, Kokkinos (2019) [54] discovered that warm parental engagement was not connected with adolescents’ relational hostile behavior and instead gave birth to hollow and weak linkages between these variables. Authoritarian parenting oftentimes does not consider and anticipate relational aggression in romantic relationships. According to Richards and Branch (2012) [55], parental social support was noted to be not directly correlated with female or male dating violence victimization. In contrast, Tyler et al. (2011) [56] reported that low parental warmth has always corresponded with dating violence perpetration and victimization, which are consistent with a social learning theory interpretation.

*Attachment Styles with Parents.* Early attachment is the foundation of self-worth and relationships. It has a substantial impact on adolescent bonds with friends, instructors, and romantic partners later in life Bowlby (1969) [20]. According to Miga et al. (2010) [41], four years later, those who had more dismissive attachment were more likely to be victimized by verbal hostility from their romantic partners. Similarly, Wright (2015) [46] discovered that separation from the young adult’s mother was associated with partner-targeted online violence. The results from this study add to our comprehension of attachment styles and their impact on adolescent romantic relationships. Fritz (2014) [39] discovered that women who were more nervously attached to their parents are more prone to be victims of online dating abuse. Furthermore, according to Simons et al. (2012) [34], children who grew up with parents who are involved in aggression are highlighted to be at the highest risk level of committing perpetration as well as being victimized. According to Santona et al. (2019) [44], father–child insecurity attachment style appears to be connected with higher levels of anxiety and avoidance in romantic relationships, followed by aggressiveness in males. On the other hand, mother–child insecurity attachment pattern appears to be associated towards higher levels of aggression within females.

*Parental Divorce.* Parental divorce is common because it is preceded by marital strife and has a negative impact on parent–child relationships; because many children may grow up in intact households with high levels of marital dispute, the effects of parents’ marital conflict in addition to parental divorce should be a primary topic of exploration [57]. Interparental conflict was found to be substantially associated with young adults’ disagreements with their partners, which were then associated with lower relationship quality. In a similar study, Bernstein et al. (2012) [49] found that parental marital status is not entirely linked with poor wellness in young adults, including unhappiness, low self-esteem, aggressiveness in romantic relationships, and romantic attachment insecurity, in a comparable study [48].

*Nonviolent Aspects of Interparental Conflict*. According to Tyler et al. (2011) [56], when parents fought more frequently, were verbally hostile during disagreements, or had poor conflict resolution, then their children were more likely to be victims of dating violence. Interparental violence was also associated with teenage perpetration and victimization of relationship violence.

Aggressive behavior in romantic relationships was identified from all the manuscripts reviewed included relational aggression [8,45], dating violence [30,31,34,43,58,59], dating aggression [29,35,36,40,41], dating violence victimization [56,60], cyber aggression within adolescents’ romantic relationships [46], and physical aggression in dating relationships [26,33]. Table 3 shows general description of study.

## 4. Discussion 

The objective of this study was to conduct a systematic evaluation of the literature on familial factors linked to aggression behavior in romantic relationships. Despite having well-established, family-level risk markers for aggressive behavior in romantic relationships, the related underlying familial variables are very much not known. Hence, we systematically evaluated various studies on familial factors in aggressive behavior in romantic relationships. Based on our research, we identified 27 eligible publications, which are diverse with regards to family characteristics, research designs, and populations examined. There were six articles that studied family violence, eight that investigated attachment styles with parents, five articles on parenting, two addressing parental divorce, four addressing interparental conflict, one addressing nonviolent aspects of interparental conflict, and one on cyber aggression in intimate relationships.

One area where there was a lack of variance in aggression was the types of aggressive behaviors examined in romantic relationships. Little research has looked at aggressive behavior in romantic relationships that is not violent or overt. Other reviews have picked up on this tendency [32,47,48]; addressing relational aggression in romantic relationships and [46] cyber aggression in romantic relationships reflects the field’s inclination to focus on non-physical partner violence. More research is required in order to draw conclusions about how familial factors are associated with nonphysical types of aggressive behavior in romantic relationship.

Based on the current review, it was known that females were noted to be more likely than males to report aggression in romantic relationships, whereas these findings could be ambiguous as a result of gender differences. According to the gender sociocultural theory, women are socialized from a very young age to think about moral concerns differently than males [44,45]. Women are also regarded to have a higher level of ethical awareness and understanding than men [46,47]. The term “aggression” was defined differently in various research. As a result, the incidence and prevalence data in the studied literature differed because of personal views and attitudes and because the level of violent behavior in love relationships varies among individuals. For researchers to acquire valid epidemiological data, they need a standardized, operational definition of aggression behavior in romantic relationships.

According to Simons et al. (2012) [34], the rate of sexual violence was underreported as a result of the broad definition of what constitutes sexual violence. Sexual coercion, for example, is considered a kind of sexual assault and occurs when the offender attempts “to get a date intoxicated or high” and can constitute “the attempt to turn a date on via caressing” although it is frequently undisclosed since the victim may not perceive it as aggressive [34]. In addition, assaulting a partner may be considered tolerable physical assault by some individuals but severe physical assault by the others. Females are more likely than males to report physical violence although males were more likely to report severe types of violence.

Attachment styles with parents are still a much-researched factor among underlying familial factors. Overall, evidence is preset to show a low to moderately significant link between these two factors and later aggression in romantic relationships. However, retrospective reporting forms the foundation of most of the evidence, and some findings imply that other proximal characteristics, such as the individual’s antisocial conduct and adult adjustment, may mitigate the relationship. As an example, the evaluation also includes studies on protecting parenting factors. Parental influences can be a positive engagement in an adolescent’s life, such as giving support and encouragement, and monitoring can help to reduce nonviolent behavior and was found to be a significantly low-to-moderate predictor of aggression in romantic relationships.

Family is the most essential socializing agent in a person’s life since it is the initial context in which the individual’s identity is formed and in which the individual interacts with others as well as the first hub between the individual and the society in which he or she lives. According to studies, parents transmit a behavioral style to their children by modelling and reinforcing certain behaviors, such as type of communication, violent behaviors, physical punishment, and parental control, that adolescents often imitate in their affective relationships, where they learn to imitate violent behaviors and attitudes already present in the family [43]. Furthermore, these parental practices have been connected to substantial behavioral and psychosocial adjustment issues in teenagers, both of which are intimately linked to couple violence. The actions and attitudes that characterize healthy family functioning are cohesion, support, and positive communication, where, on the other hand, are linked to improvement in children’s psychological and emotional development.

Family violence is still one of the most-studied familial factors. Overall, there is evidence of a low to moderately significant relationship between family violence and aggression behavior in romantic relationships. However, much of the evidence was based on retrospective reporting, and limited findings suggested that more proximal factors, such as antisocial behavior and adult adjustment, may mediate the association. According to the Lee et al. (2013) [43] study, the effects of family violence, like other early risk factors, appear to be mediated by subsequent problematic development, such as antisocial behavior and substance-use problems. There is no evidence of significant gender differences in these associations.

Across studies, the methods employed to retrieve data on aggression behavior were generally the same. The CTS2, on the other hand, was not introduced to gather data in regards to how such assault happens [18]. The CTS2 examines about both mild and major kinds of physical violence. However, it fails to take into account about the commencement, intent, or background of partner violence [18]. As a result of vast concentration on the amount of aggression behavior acts without accounting for the features of aggressive behavior between couples, the rate of perpetration reported may be deceptive [18].

In the last eleven years, significant strides were made in studies investigating the familial influences and aggressive behavior in romantic relationships, with a greater emphasis on aggressive behavior in romantic relationships or dating violence in adolescents and adults. A large number of the studies were longitudinal, which is a significantly better strategy for identifying familial influences than cross-sectional studies. Overall, the findings on familial factors for aggressive behavior in romantic relationships were strikingly similar to those for risk factors for other adolescent and adult problems involving risky behavior, such as crime, substance use, and sexual risk behavior, implying that aggressive behavior in romantic relationships is theoretically similar to this behavior. This has significant implications for research, particularly in the creation of treatments or prevention strategies to reduce aggressive behavior in romantic relationships. Table 4 refers to the findings of studies.

## 5. Limitations and Future Directions

Despite its many positive features, this review has several shortcomings that can be addressed in order to further the study into underlying familial characteristics and aggressive behavior in romantic relationships. Several pieces of the reviewed research relied on the self-reporting of parental attachment, partner attachment, relational victimization, and also digital violence in romantic relationships. As a result of this, self-report biases were identified as a concern in this review. Hence, in order to overcome this concern, follow-up studies should include reports from both parents and partners. Partner-based reports may be extremely useful in comprehending partner-directed aggression because youth in romantic relationships may have under-reported these behaviors to maintain a good self-image. Peer nominations appear to be a better predictor of aggressiveness in romantic relationships among early young adults in comparison to self-reports; therefore, this is a field that should be further explored [63]. Extensive research is also needed to provide a complete grasp of how various and multiple familial circumstances contribute to aggressive behavior in romantic relationships.

Furthermore, overcoming self-report biases will aid in comprehending partner-directed aggression in romantic relationships when it is reciprocated as well as offering more information on the nature and scope of these behaviors. Both couples should report romantic relationship attachment. People typically choose partners who have comparable attachment styles to themselves [64]. Individuals with similar parenting styles or attachment styles with parents might demonstrate different associations with aggressive behavior in romantic relationships than partners with different parenting styles or attachment styles. 

Parental attachment is steady across time though it might improve or deteriorate depending on the parent–adolescent relationship’s current situation. Therefore, follow-up research is necessary to evaluate parental attachment at several parts of a linear time horizon in order to gain a better grasp of the relationship between this type of attachment and aggressive behavior in romantic relationships. Further, it is also likely that this review left out some important studies on the subject. Only peer-reviewed works published between 2010 and 2021 met the review requirements. We focused on papers published after 2010 to capture current family factors and aggression behavior in romantic relationships; however, some pertinent studies may have been missed. In addition, as a result of excluding gray literature in our search technique, we note that the review may not have been totally thorough within the 11-year timeframe.

The cross-sectional character of the data, which prevents judgments about causal correlations, is another weakness seen in all of the investigations. Furthermore, given that the majority of the participants in the peer-reviewed publications are a homogeneous group of college students, caution should be practiced when extrapolating the findings to young men and women outside of institutional establishments. The current study’s findings are further hampered by the use of the most often-used instrument (Conflict Tactic scale: CTS2) to measure violent perpetration and victimization because it does not examine the contextual nature of violence.

In particular, the CTS2 lacks markers that would indicate if reported acts of aggressiveness are reactive behaviors used in self-defense. Moreover, one of the limitations in this study is the inclusion of all forms of familial factors rather than focusing on a specific familial factor, and it is possible that some key papers on the issue were overlooked in this review because we used a broad search term in this review. Hence, future research should focus on a specific familial factor that would allow researchers to provide a better and more meaningful conclusion. Finally, while this study contends no generalizability to dating couples in general, the findings from our review, which shed light on the familial factors that underpin aggressive behavior in romantic relationships in this emerging adult population, can be used to guide the development of more effective prevention interventions for aggressive behavior in romantic relationship and promotion of healthier intimate relationships in later life. 

## 6. Conclusions

According to our systematic review analysis, there is no evidence that specific familial factors are linked to aggressive behavior in romantic relationships among adolescents and emerging adults. The level of methodological rigor and modeling methodologies limit our conclusions. Familial factors contributing to aggressive behavior in romantic relationships should be identified in order to formulate effective aggressive-behavior-prevention measures in romantic relationships. It was known that approximately two-thirds of marriages ending in divorce; hence, it is no surprise that experts are focusing their efforts on figuring out what makes a relationship healthy [65]. Regarding the relevance to relationship functioning, aggressive behavior in romantic relationships has gained widespread attention, and it has many consequences for clinical therapy for victims. As evidence suggests, familial factors have a negative impact on aggressive behavior in romantic relationship functioning, and a thorough assessment of past victimization experiences and related suffering among clients, particularly in couples therapy settings, will be beneficial. Furthermore, depending on the nature of the client’s presenting issues, clinicians may or may not be focused on the aggressive behaviors in which clients may engage within their romantic relationships as well as their relationship functioning concerns. However, many people, particularly those with a history of family violence, may find these issues to be significant. As a result, clinicians may assess for family violence and relationship issues and if necessary, incorporating these targets into treatment planning. Effective parenting skills and family social support have been related to a lower risk of aggressive behavior in romantic partnerships, and efforts to improve collective effectiveness may help to reduce aggressive behavior in romantic relationships. As a result, focusing on current relationship experiences offers a one-of-a-kind and crucial opportunity for people who have been victimized in the past to change implicitly and potentially destructive relationship expectations. Our research has thus far emphasized the importance of instilling knowledge about fostering positive pair interaction for those may be more vulnerable to aggressive behavior in their existing romantic relationships, and we believe that the findings of this study give excellent evidence for designing a variety of prevention programs aimed at aggressive behavior in romantic relationships.

## Figures and Tables

**Table 1:** Keyword Search List.

Term	Keyword That Was Used for Searching Process
Familial Factors	(“Familial Factors” OR “Family Support” OR “Family Relationship”) AND
Aggressive Behavior	(“Aggressive Behavior” OR “Aggression” OR “Violent”) AND
Romantic Relationship	(“Intimate Relationship” OR “Adult” OR “Love”)

**Table 2:** Article-Finding Criteria.

Criteria	Inclusive	Exclusive
Year Duration	2010–2021	No exclusion
Language	English/Malay article	Not English/Malay articles
Country	All countries	No exclusion
Article	Type of Journal (Empirical Data)	Not a research article

**Table 3:** General Description of Study.

References	Country	Design	Sample Size	Familial Factors	Aggressive Behaviors in Romantic Relationships
Gover et al. (2010) [14]	USA	Cross-sectional study	A mean average of 24 years (SD = 3.20) from a total of 2541 students.	Violence in the family of origin	Dating violence among college students
Milletich et al. (2010) [26]	USA	Cross-sectional study	Participants (183 males, 475 females)	Exposure to interparental violence and childhood physical abuse	Physical aggression in dating relationships
Fosco et al. (2016) [28]	USA	Cross-sectional study	Young adults (*n* = 974; M = 19.5).	Family relationship quality as key individual and family-level factors	Adolescent hostile aggressive behavior
Karakurt et al. (2013) [29]	USA	Cross-sectional study	The sample consisted of 87 heterosexual pairs with an average age of 22.3 years (SD = 4.80).	Parental conflict and parent to child aggression	Dating aggression
Laporte et al. (2011) [30]	Canada	Cross-sectional study	A total of 471 teenagers aged 12 to 19 were recruited from a population sample and those in the care of a juvenile protection agency.	Family violence	Dating violence
Byrd and Bierman (2013) [31]	USA	Longitudinal study	Individuals (*n* = 401, 43 % were female) were followed from preschool level to the age of 18 years.	Family factors	Dating violence
Paat et al. (2016) [34]	USA	Cross-sectional study	The sample for the study included 3495 participants ranging in age from 18 to 40 years old from several (16) public, rural, private, urban, or suburban universities or colleges across the USA.	Family social structure	Physical aggression in dating
Simons et al. (2012) [34]	USA	Cross-sectional study	2088 undergraduates (1774 women and 314 men)	Parental warmth	Dating violence
Tschann et al. (2010) [35]	USA	Cross-sectional study	150 male and female adolescents, aged 16 to 20.	Nonviolent aspects of interparental conflict	Physical violence and verbal aggression in romantic relationships
Vezina et al. (2015) [36]	Canada	Cross-sectional study	443 female individuals were assessed during adolescence (age 15) and early adulthood (age 21).	Influence of history of family violence	Dating victimization; psychological, physical, and sexual violence
Xia et al. (2018) [37]	USA	Cross-sectional study	974 pre-adolescents (mean age = 12.4, 62.1% female) were tracked through to young adulthood (mean age = 19.5).	Parenting practices	Romantic relationship violence
Clarey et al. (2010) [38]	USA	Cross-sectional study	204 high-school students of ages 15 to 17 from Mexico, in which 129 were female, and 75 were male.	Interparental conflict	Dating violence among teens
Fritz et al. (2013) [39]	Canada	Cross-sectional study	137 heterosexual female college students (18 to 25) with mean of 20.76 (SD = 1.87).	Attachment style with parents	Dating aggression
Grych and Kinsfogel (2010) [40]	USA	Cross-sectional study	391 students aged 14 to 18 years old, in which 52% of them were female, with mean age of 15.6 and SD of 1.1 years.	Family aggression	Dating aggression
Miga et al. (2010) [41]	USA	Longitudinal study	93 adolescents who had romantic partners (1st quarter mean = 14.28, SD = 0.78); (2nd quarter mean = 18.25, SD = 1.25). 42% male and 58% male.	Paternal attachment insecurity	Romantic partner’s aggression
Lee et al. (2013) [43]	USA	Cross-sectional study	A total of 351 men, or about 8% of the overall sample, took part in the study.	History of childhood family violence	Male intimate partner violence
Santona et al. (2019) [44]	Italy	Cross-sectional study	There were 43 females and 168 males. The students ranged from age 14 to 18, with a mean age of 16.85 years and SD of 1.41.	Attachment styles with parents	Aggression in romantic relationships
Clark et al. (2015) [45]	USA	Cross-sectional study	323 students consisting of 89 males and 234 women (median age = 19) from a midsized U.S. university took part in the study.	Parenting styles	Relational aggression
Wright (2015) [46]	Greece	Cross-sectional study	Total participants were 600 (326 female with mean age = 17.53, SD = 0.51) 12th graders from one public high school.	Parental attachment	Cyber aggression in romantic relationships
Kokkinos and Voulgaridou (2017) [47]	Greece	Cross-sectional study	261 Greek junior-high school children comprising of 127 girls of varying age (12 to 15) (SD = 1.22, mean = 13.4).	Parenting behaviors	Relational aggression
Bernstein et.al (2015) [49]	USA	Cross-sectional study	Participants were 45 college students amounting to 60% from a prominent public university in California; 37 of them were women. (mean age = 20.6, SD = 2.3).	Parental divorce	Aggression in romantic attachment
Heifetz et al. (2010) [50]	Canada	Cross-sectional study	1765 young teenagers (grades 5 to 8) from intact and divorced homes; each 1315 and 379.	Parental divorce, family conflict, and parental monitoring	Adolescent conflict relationships
Richards and Branch (2012) [55]	USA	Longitudinal study	Wave I conducted in 2001 of the Toledo Adolescent Relationship Study (TARS), a 5-year investigation (*n* = 970 participants).	The level of parental social support	Physical dating violence
Tyler et al. (2011) [56]	Canada	Cross-sectional study	80 male and female high-school students as well as 52 middle-school students from several parts of the United States participated.	Effect of poor parenting	Male and female dating violence
Leadbeater et al. (2017) [60]	Canada	Longitudinal study	Six cycles of data involving 662 participants (342 females) aged 12 to 18 years old in 2003 were collected biennially during a ten-year period. This study focused on 334 youth who were in a current romantic relationship at the sixth phase (T6, 10 years later).	Parents psychological control	Intimate partner violence
Berzenzki et al. (2010) [61]	USA	Cross-sectional study	A total of 2169 undergraduate students (63.8% female) from a large West Coast university took part in the study.	Childhood emotional abuse	Relationship violence
Lohman. et al. (2013) [62]	USA	Cross-sectional study	19 to 23 years and adults from 27–31 years according to the Iowa Youth and Families Project (*n* = 392; 52% of female).	Parents’ psychological violence	Intimate partner violence

**Table 4:** Findings of Studies.

Reference	Tool	Finding	Limitations
Gover et al. (2010) [14]	Revised CTS (CTS2; Straus, Hamby, Boney McCoy, & Sugarman, 1996)	When compared to those who did not observe their mother hitting their father, students who experienced such violence are more likely to endure physical violence in romantic relationships. In dating relationships, women are more likely than males to be the victims of physical abuse.	The cross-sectional character of these data, judgments concerning causal links are impossible to draw. Furthermore, given the participants in this study were a diverse group of students, caution is required when extrapolating the findings to individuals outside of the academic in situations.
Milletich et al. (2010) [26]	Revised Conflict Tactics Scale (CTS2-CA; Straus 2000) Exposure to Abusive and Supportive Environments Parenting Inventory (EASE-PI; Nicholas and Bieber 1997)	The extent of dating aggressiveness was linked to exposure of mother-to-father violence and constant childhood abuse among women. These forms of abuse as a youngster were linked to the severity of dating aggression in men.	Future studies may include the verification of these findings by surveying both dating partners, obtaining parental accounts, and doing experimental investigations that directly monitor partner’s actions.
Fosco et al. (2016) [28]	Hostile/Aggressive Behaviors scale, which was derived from the National Youth Survey (Elliott, Huizinga, and Ageton, 1985) Family Environment Scale (Moos and Moos, 1994) Love and Conflict Scale (Braiker and Kelley, 1979) Conflict Tactics Scale (Strauss, 1979) Aggression Questionnaire, adapted from Buss and Perry (1992)	Reduced hostile aggressive behavior was linked to a more favorable family climate, but hostile aggressive behavior was not linked to changes in family climate. Furthermore, the impact of the family climate on hostile aggressive behavior remained stable throughout time. Young adult romantic relationships were predicted differently by hostile aggressive behavior and familial climate: increased HAB during adolescence predicted relationship violence, whereas family stability predicted relationship violence.	This study was confined to data from a single informant, which may have skewed or exaggerated the degree to which certain factors are connected. Multi-informant approaches to analyzing familial, individual, and romantic relationships as well as multimethod techniques that depend on objective assessments should be used in future studies.
Karakurt et.al., (2013) [29]	Parent-to-Child Violence (Cappell and Heiner 1990; Kwong et al. 2003) Conflict Tactics Scale (CTS2; Straus, Hamby, Boney McCoy, & Sugarman, Straus et al. 1996) Emotional Abuse Questionnaire (EAQ; Jacobson and Gottman 1998)	Women who had witnessed parental conflict were more likely to be mistreated by their spouses. Parental conflict had a strong favour able direct influence on women who were assaulted by their partners when they were children.	It’s critical to expand this research by gathering more data from a wider spectrum of individuals in terms of age and socioeconomic background.
Laporte et al. (2011) [30]	The Sexual and Physical Abuse Questionnaire (SPAQ) Conflict Tactics Scale (CTS; Straus, 1979) The Conflict in Relationships scale (CIR; Wolfe, Reitzel-Jaffe, Gough, & Wekerle, 1994)	Depending on gender and risk level, adolescents transfer negative childhood experiences of family violence into their intimate relationships in various ways. Female adolescents who had been abused by either of their parents were more likely to experience victimization in their dating relationships but not aggression. Adolescent boys who had experienced childhood trauma were more likely to be aggressive toward their girlfriends, especially if their father had reprimanded them brutally.	The variability within the high-risk group, as seen by the lack of access to the adolescents’ partners’ perspectives, is one of these results: these adolescents were referred to protective services for a variety of issues.
Byrd and Bierman (2013) [31]	Conflict Tactics Scale (CTS; Straus 1979) Developmental History questionnaire (Dodge et al. 1990; Laird et al. 2003) The Externalizing Broadband Raw Score of the Child Behavior Checklist (CBCL)—Parent Report Form (alpha = 0.91) (Achenbach, 1991)	The findings show that aggressive family dynamics in childhood and early adolescence promote the development of dating violence by encouraging a child’s oppositional-aggressive reacting style in the home, which is subsequently generalized to other situations.	The contribution of longitudinal evidence, including parent, teacher, and teenage accounts from both boys and girls, is restricted by flaws mentioned in the discussion. A study on the correlation between variables and person-oriented group comparisons as well as a dual-emphasis on the prediction of perpetration and victimization combined would create a distinctive contribution to the burgeoning literature.
Paat et al. (2016) [33]	Conflict Tactics Scales (CTS2) Household structure and their parents’ educational attainment were proxies for the participants’ family social structure	Although connections with parental education were not mathematically significant, participants were more likely to undergo victimisation or inflict aggression in relationships characterised by conflicts, distress, dominance, or psychological aggression when they lived in a two-parent household.	Examining relationships and interactions with other variables could lead to hypotheses for future longitudinal investigations. Furthermore, students are more likely to reflect middle-class communities. Hence, violence in the college context can be different qualitatively from violence in another set of samples with similar interest. Furthermore, due to fear, some participants may have been hesitant to disclose any involvement in violent misconducts.
Simons et al. (2012) [34]	Conflict Tactics Scale (Straus, 1990) Harsh and supportive marital interaction scales: Conger and Elder (1994) Iowa Youth and Families Project (IYFP; Conger & Elder, 1994; Conger, Elder, Lorenz, Simons, & Whitbeck, 1992)	Interparental hostility interacted with interparental friendliness to predict dating violence in both men and women. Maternal warmth mitigated the effect of maternal hostility on dating violence in women, such that the link between maternal hostility and both markers of dating violence (perpetration and victimization) was much larger when the mother also displayed warmth.	Because the findings appear to be one of the pioneers to show such amplification effects, they must be replicated.
Tschann et al. (2010) [35]	Multidimensional Assessment of Interparental Conflict (MAIC; Tschann, Flores, Pasch, & Marin, 1999) Psychological Aggression and Physical Assault subscales of the Revised Conflict Tactics Scale (CTS2; Straus, Hamby, BoneyMcCoy, & Sugarman, 1996)	At the 12-month follow-up, 77 percent of teenagers admitted to engaging in or attempting to engage in sexual activity. Their most recent dating partners have been verbally abusive. Interparental violence was found to be a strong predictor of relationship violence. The findings suggest the importance of nonviolent parental conflict as a factor in adolescent dating violence, in addition to the effects of interparental violence.	The findings are not suitable to be applied to teenagers whose parents are either divorced or not insured, as they aremore likely to be involved in dating related violence. Furthermore, those who were not able to follow-up may have been at a higher threat of being involved in dating violence.
Vezina et al. (2015) [36]	Psychological Maltreatment of Women Inventory (PMWI; Kasian & Painter, 1992) Conflict Tactics Scale (CTS; Straus, 1979) Sexual Experience Survey (SES; Koss & Gidycz, 1985)	A higher likelihood of girls being victimised mentally and/or physically/sexually in their romantic relationships was linked to a history of parental violence, childhood behavior is sues, and teenage high-risk behaviors, whether in adolescence, early adulthood, or both developmental stages.	Reproduce this study with a bigger and more varied sample to enhance the findings so that it is more universal. Finally, the reference period employed in the dating victimisation measures which refers to a specific time period of 1 year and just one love partner could be linked to an underestimating of victimisation rates.
Xia et al. (2018) [37]	Family Environment Scale (Moos and Moos, 1981) General Child Management Scale (Spoth et al. 1998) Love and Conflict Scale (Braiker and Kelley, 1979) Cooperative Problem Solving Measure (Assad et al. 2007) Conflict Tactics Scale (CTS; Straus, 1979)	In their young adult love relationship, adolescents who had grown up in a more pleasant familial environment reported better problem-solving skills and less violent behavior. Positive adolescent family interaction was linked to feelings of love in young adult romantic relationships.	Future studies may consider to extend the cognitive development scope further into early childhood. This would in return provide a more comprehensive view of the development of young adult romantic relationships. Besides, other relationship outcomes can be examined andemulate these observations with multi informant mixed methodology participant, mixed methodology information would be favourable.
Clarey et al. (2010) [38]	Exposure to Interparental Conflict Interparental conflict Moos and Moos (1994) Conflict in Adolescent Dating Relationships Inventory (CADRI) Wolfe et al. (2001). Acceptance of Couple Violence Foshee, Fothergill & Stuart (1992)	Anger management, interparental conflict exposure, and dating violence perpetration are all linked in a substantial way. Acceptance of violence, exposure towards interparental and dating violence perpetration were also found to be significantly associated to each other, with those who experienced significant amount of interparental violence and tolerate the use of violence in their dating relationships being the most likely	The findings suggest the use of family based therapies for Mexican youth and their parents that address inter-parental conflict, highlight anger management skills, and challenge acceptance of violence ideas.
Fritz et al. (2013) [39]	The Experiences in Close Relationships Scale (ECR; Brennan et al., 1998) The Conflict in Adolescent Dating Relation ships Inventory (CADRI; Wolfe et al., 2001)	If women were more anxiously linked to their parents, they reported greater incidence of dating victimisation (r = 0.30, *p* = 0.000).	The researchers made no distinction between women who just reported victimisation and those who reported both victimisation and DA perpetration. Women that are mutually aggressive should be compared with women that are victims only to have a deeper clarification of the risk factors connected with DA victimization.
Grych & Kinsfogel (2010) [40]	Conflict Tactics Scale (CTS; Straus, 1979) The Conflict in Relationships scale (CIR; Wolfe, Reitzel-Jaffe, Gough, & Wekerle, 1994) Attitudes About Dating Index (AADI; Foo & Margolin, 1995)	By altering their ideas about the acceptability of aggressiveness and their ability to control and manipulate anger, youths’ romantic attachment style might enhance the effects of familial aggression on abusive behavior in dating relationships.	Because the data is cross-sectional, it can not be used to address causation concerns. It’s unclear if youngsters’ job framework influence their behavior toward their partners or vice versa. This is due to that the nature of their relationship affects their attachment style. Longitudinal research would allow for a more sensitive examination of the relationship between attachment, attitudes, emotions, and behavior.
Miga et al. (2010) [41]	Adult Attachment Interview (George, Kaplan & Main, 1996) and Q-set (Kobak, Cole, Ferenz-Gillies, Fleming & Gamble, 1993) Psychological Maltreatment Experience Scale (PMES) (Petretic-Jackson, Betz & Pitman, 1995) Conflict in Relationships Scale (CIR) (Wolfe, Reitzel-Jaffe, Gough & Wekerle, 1994)	After accounting for other attachment indices acquired from the teen, measures from the Experience in Close Relationships questionnaire (partner report) showed that teen attachment anxiety remained predictive of teen physical aggression. In the romantic context, overall attachment insecurity increases the chance of perpetrating aggression.	Although the use of a community sample maximises the accurateness of the findings, partner aggressiveness was typically mild, therefore these findings should not be applied to high-risk populations.
Lee et al. (2013) [43]	Aggression Questionnaire, adapted from Buss and Perry (1992) Kurdek’s (1994) Destructive Arguing scale Hamby (1996) Dominance scale	According to the findings, perpetrators with a history of family violence are more likely to support notions that portray women and feminine characteristics in a negative light. While childhood exposure to familial violence is not required for IPV to develop, it may be a marker for more severe attitudinal and behavioral disorders.	The study relied on men’s self-reports, which could have resulted in reporting bias. This is especially true in the case of retroactive claims of family violence, which are prone to recall limits or intetional misreporting due to social desirability.
Santona et al. (2019) [44]	The Inventory of Parent and Peer Attachment (IPPA) (Greenberg et al. 1983). The Aggression Questionnaire (AQ) (Buss and Perry, 1992) The Attachment Style Questionnaire (ASQ) (Feeney et al. 1994)	Insecure father-child attachment styles in males seems to be linked to higher levels of anxiety and avoidance in romantic relationships, followed by aggressiveness. An insecure mother-child attachment pattern appears to be linked to greater levels of aggression in females.	Because a battery of instruments was given to all of the participants at the same time, the findings may be tainted by a social desirability bias. A stratified analysis based on age class, as well as a deeper evaluation of the diversification in the parameters included in this study and other sociocultural characteristics, might be possible with more research with a bigger sample size
Clark et al. (2015) [45]	Parental Authority Questionnaire (PAQ; Buri, 1991) Self-Report of Aggression and Social Behavior Measure (SRASMB; Morales & Crick, 1998)	Relational aggression was found to be negatively connected to authoritative parenting, implying that emerging adults who were raised in this fashion were less likely to engage in relationally hostile activities. Permissive parenting and parental psychological control, on the other hand, were positively linked to relational aggressiveness.	As means for continuous learning towards the formation and maintenance of relational aggressiveness, researchers must look into the temporal and reciprocal patterns of the constructs.
Wright (2015) [46]	Self-reported Partner-Directed Cyber Aggression The inventory of parent and peer attachmen (Armsden and Greenberg 1987)	Through the mediation of anxious partner attachment, insecure parental attachment from teenagers’ mothers was linked to insecure partner attachment and had an indirect effect on their relationship-directed cyber violence.	In terms of parental attachment and partner attachment as well as cyber violence in romantic relationships, the current study relied on self-reports. Self-report are generally bias. Hence, follow-up studies should include both parent and partner reports due to the fact that adolescents may have underreported these actions so that a favourable self-presentation can be preserved. The partner report may be useful for learning more about relationship-directed cyber violence
Kokkinos & Voulgaridou, (2017) [47]	The five-item RA subscale from the children’s social behavior scale-self report (CSBS-SR; Crick & Grotpeter, 1995) 26-item parenting styles questionnaire (PSQ; Kokkinos et al., 2016; Lamborn, Mounts, Steinberg, & dornbusch, 1991)	The findings supported the hypothesis that parental psychological control (the polar opposite of psychological autonomy) would be positively correlated with Relational Aggression, confirming previous research findings that adolescents with more psychologically controlling parents are more likely to engage in relationally aggressive behaviors in roman tic relationships.	Although the sample’s size and cultural variety are significant, a college student sample’s selection to the overall population is limited. Despite the sample’s small age bracket, it represented an age cohort with a high risk of dating violence (Capaldi, Shortt, & Kim, 2005; Wilt & Olson, 1996), making it more scientifically relevant than a sample of different ages.
Bernstein et al. (2015) [49]	Experiences in Close Relationships-Revised Scale (ECR-R; Fraley, Waller, & Brennan, 2000) Children’s Beliefs about Parental Separation Scale (CBAPS; Kurdek & Berg,1987)	Parental divorce, and particularly the fear of abandonment, increases the chance of insecure romantic attachment, but not of other bad outcomes.	Gauge the relationships presented here with a bigger data sample which would give you the potential to look at moderators like age at divorce and a more evenly distributed gender distribution.
Heifetz et al. (2010) [50]	Conflict Resolution Scale was adapted from Parker and Asher’s (1993) Strictness-Supervision Scale (Steinberg, 1989) Armsden and Greenberg’s (1987) Inventory of Parent and Peer Attachment (IPPA)	In comparison to young adults from intact families, teenagers from divorced homes report more dating, are more susceptible to romantic influence, and have similar romantic relationship quality.	Future research should keep a developmental focus because adolescents may react differently to contextual elements within the family system. In addition to the variables explored in this study, other variables should be investigated because family processes may play a vital influence in teenagers’ romantic relationships
Richards & Branch (2012) [55]	Revised Conflict Tactics Scale (Straus & Gelles, 1990)	Parental social support has little bearing on whether a child is a victim of dating violence. Guys who are older, have experienced more parental violence, and have higher average grades are more likely to be victims of dating violence than younger males who have experienced less parental abuse. When comparing male kids with greater levels of teenage committed family violence to male youth with lower levels of teenage perpetrated family violence, dating violence victimisation is lower	To examine the temporal ordering of individuals’ degrees of social support from family and friends and their involvement in dating violence, longitudinal studies are needed. The current study omitted a measure of emotional dating violence, which, according to previous research, can exist in relationships even when there is no physical violence.
Tyler et al. (2011) [56]	Lack of parental warmth was measured at Wave 1 and included six items regarding the respondent’s relationship to his or her residential mother and residential father. Dating violence perpetration was measured at Wave 3 using three items that asked respondents about their violent behaviors toward a dating partner. Dating violence victimization was measured at Wave 3 using three items that asked respondents about their partner’s violent behaviors.	More physical abuse, poor parental warmth, and increased delinquency all had positive direct impacts on dating violence perpetration, while more physical abuse, low parental warmth, and higher delinquency all had positive direct impacts on dating violence victimisation.	First, respondents were asked to report on their spouses’ violent behavior toward them, which may have led to some underreporting. Second, because the data set contained few distinct signs of violence, we were unable to keep the outcome variables continuous, which may have limited our capacity to explain dating violence.
Leadbeater et al. (2017) [60]	The Self-Report Measure of Dating Victimization and Aggression (Leadbeater et al., 2008; Linder et al., 2002) Psychological Control Scale Youth Self-Report (PCS; Barber 1996)	In adolescence and early adulthood, experiences of parental psychological control and relational aggression due to peer pressure as well as victimization promote young adults’ tolerance for, or use of, relational intimate partner violence as a method in resolving romantic relationship issues.	Multiple variables that have been linked to physical IPV in previous studies must be examined. We also excluded physical aggression and victimization from romantic partnerships, so we cannot draw any conclusions about potential links between physical and relational aggression in romantic partnerships.
Berzenski et al. (2010) [61]	The Childhood Maltreatment Interview Schedule (Briere, 1992) Difficulties in Emotion Regulation Scale (DERS; Gratz & Roemer, 2004) Reactive Anger scale of the State–Trait Anger Scale (STAXI; Spielberger, Jacobs, Russell, & Crane, 1983) The Conflict Tactics Scale (CTS; Straus, 1979)	Both victimisation and perpetration of marital violence were predicted by childhood emotional maltreatment.	The study’s exclusive focus on dating aggression, longitudinal data is still being gathered on this sample, which will likely result in a larger sample of teen daters over time, allowing for prospective assessments of the relationships examined in this study.
Lohman et al. (2013) [62]	Self-Efficacy in Romantic Relationships (SERR; Bradbury, 1989) Children’s Perceptions of Interparental Conflict Scale (CPIC; Grych, Seid, & Fincham, 1992)	The findings reveal that adolescent exposure to psychological violence from parents is a strong predictor of intimate partner violence in adulthood. In adulthood, exposure to family stress was linked to intimate partner violence, but not in emerging adulthood.	The study’s cross-sectional design limits the capacity to assess directional hypotheses about causation and effect. Given this framework, it is impossible to conclude that emotional abuse leads to difficulties with emotion control, which, in turn, contributes to interpersonal violence.
